# A Comparison of Postoperative Urological Infection Rates Between Supine and Prone Positions During Percutaneous Nephrolithotomy

**DOI:** 10.5152/tud.2025.25024

**Published:** 2025-05-21

**Authors:** Nattanachoti Batratanakij, Chatchawet Liwrotsap, Teerayut Tangpaitoon

**Affiliations:** Department of Surgery, Thammasat University Hospital, Pathum Thani, Thailand

**Keywords:** Complications, PCNL, percutaneous nephrolithotomy, postoperative urological infection, prone position, supine position

## Abstract

**Objective:**

:Urological infection is a significant complication following percutaneous nephrolithotomy (PCNL), which can be performed in supine or prone positions, but its impact on infection rates remains debated. This study compares postoperative urological infection rates between supine and prone PCNL and identifies associated risk factors.

**Methods:**

:A retrospective study was conducted on 290 patients who underwent PCNL between January 2014 and August 2023 in the Thammasat University Hospital. Patients were allocated into 2 groups in a 1 : 2 ratio, with 87 patients in the supine group and 203 patients in the prone group. Statistical analysis included *t*-tests, Mann–Whitney *U* tests, Fisher’s exact test, and logistic regression models.

**Results:**

:Postoperative infection rates were significantly lower in the supine group, including fever (33.3% vs. 62.1%, *P* < .001), UTI (11.5% vs. 32.5%, *P* < .001), and sepsis (6.9% vs. 17.2%, *P* = .021). Multivariable analysis identified positive preoperative urine culture (RR 4.41, *P* < .001) and prone positioning (RR 4.38, *P* = 0.004) as independent risk factors. The operative time was significantly shorter in the supine group (103.9 ± 42.6 vs. 116.3 ± 38.9 min, *P* = .016). Stone-free rates and blood loss were comparable, whereas pleural complications were higher in the prone group (6.4% vs. 0%, *P* = .016).

**Conclusion:**

Supine PCNL is associated with significantly lower postoperative infection rates, shorter operative times, and reduced pleural complications compared to the prone position. These findings support supine PCNL as a safer and equally effective alternative management for renal calculi.

**Main Points**

- The supine group showed significantly lower rates of postoperative infections, including fever, urinary tract infection, and sepsis compared to the prone group.
- Positive preoperative urine culture and prone positioning were identified as independent risk factors for postoperative infections.
- The supine position was associated with shorter operative times compared to the prone position.
- Stone-free rates and blood loss were similar between the supine and prone groups.

## Introduction

Urolithiasis is a common problem worldwide with potential complications, including back or flank pain, hematuria, pyelonephritis, and chronic kidney disease. Currently, the standard treatment for renal stones larger than 2 centimeters or complicated renal stones is percutaneous nephrolithotomy (PCNL),^1^ introduced by Fernstrom and Johansson in 1976 in a prone position.^2^ However, this positioning often presents cardiopulmonary complications and difficult airway management during surgery, particularly for obese patients. Additionally, complications may include bleeding, pneumothorax, pleural effusion, injuries to the liver, spleen, or bowel, ocular injuries, and urological infections.

In 1987, Gabriel Valdivia^3^ developed a surgical approach in a supine position. Subsequent studies have shown improved surgical outcomes, such as improved stone-free rates (SFRs),^4^ reduced time for patient positioning, shorter operative time, decreased radiation exposure, less fatigue for the surgeon, easier airway management, and the ability to perform bilateral surgeries simultaneously.^5^^,^^6^ Mounting studies have also indicated that this approach may reduce complications, such as pneumothorax, pneumonia, injuries to the liver, spleen, or bowel, and post-operative infection.^7^^-^ ^9^

Postoperative infections are a significant complication leading to increased morbidity and mortality, extended hospital stays, longer courses of antibiotics, and higher healthcare costs. Many studies have reported the infection rate of PCNL. Risk factors for postoperative infection include a positive preoperative urine culture, staghorn calculi or infected stones, placement of a nephrostomy tube or ureteral stent, diabetes, stone burden larger than 800 square millimeters, and intrarenal pressure exceeding 30 mmHg.^10^^-^ ^13^ Each of these studies has provided definitions of infection based on Rangel–Frausto’s criteria established in 1995, which defined systemic inflammatory response syndrome.^14^ According to the Sepsis-3 consensus in 2016,^15^ the revised definitions for infection aim to enhance precision in diagnosis and treatment planning.

Currently, there is no clear consensus about positioning during PCNL^1^ due to many influencing factors, including stone size, number, location, renal anatomical abnormalities, and surgeon’s preference. Recognizing these issues, the researcher designed this study to compare the rates of urological infections following PCNL performed in the supine versus prone positions, aiming to provide data for selecting the most appropriate approach for individual patients.

## Material and Methods

### Objective

#### Primary Objective:

To study the postoperative urological infection rates following PCNL between supine and prone positions.

#### Secondary Objective:

To evaluate risk factors associated with postoperative urological infection following PCNL in both supine and prone positions.

### Study Design

The retrospective study reviewed patients who underwent PCNL between January 2014 and August 2023 in the Thammasat University Hospital. Patients aged over 18 years old with renal calculi or renal pelvic calculi larger than 2 centimeters, along with concomitant endoscopic management such as endoscopic combined intra-renal surgery (ECIRS) or simultaneous bilateral endoscopic surgery (SBES), were included in the study. Exclusion criteria were renal anatomical abnormalities, uncontrolled coagulopathy, untreated urinary tract infection (UTI), pregnancy, a potential malignant renal tumor, and the presence of pus after renal puncture.

The Human Research Ethics Committee of Thammasat University (Medicine) MTU-EC-SU-1-165/66 approved this study protocol, and informed consent was obtained.

### Sample Size

The sample size was calculated based on the pilot study, which included data from 30 patients in each group. The infection rate was found in 2 patients (7%) in the supine group, and 7 patients (21%) in the prone group, with an allocation ratio of 1 : 2 between the supine and prone groups. Consequently, a minimum of 67 patients was required for the supine group and 134 patients for the prone group.

### Statistical Analysis

Initially, patients were divided into 2 groups: supine and prone. For normally distributed data, *t*-tests were used, with results presented as mean ± standard deviation. For non-normally distributed data, results were expressed as median (interquartile range; IQR) and analyzed using the Mann–Whitney *U* test. Categorical data were compared using Fisher’s exact test. Subsequently, all risk factors that caused urological infection were identified through univariable logistic regression; those with a *P*-value <.2 were included in multivariable logistic regression. Statistical significance was determined with a *P*-value <.05 and a 95% CI for the risk ratio (RR). Statistical analysis was calculated using STATA software version 15.1 (Stata Corp., College Station, TX, USA).

### Data Collection

Data were collected, including age, gender, body mass index (BMI), underlying medical disease, hemoglobin levels, hematocrit, urine analysis, urine culture, stone burden, stone location, stone attenuation, surgical position, operative time, renal access, tract size, choice of renal drainage, blood loss, any intraoperative complications, residual stone, SFRs, stone composition, any postoperative complications (such as bleeding, pleural injury, bowel injury, and urological infection), hemoglobin levels, hematocrit, urine analysis, urine culture, hemoculture, stone culture, and the complications as reported in the Modified Clavien-Dindo classification.^16^

Patients with positive urine cultures were treated with pathogen-specific antibiotics (oral antibiotic for 7 days, intravenous antibiotic for 3 days, or until sterile urine). A prophylactic antibiotic was administered within 30 minutes before surgery.

Urological infections following PCNL were defined as fever (body temperature >38°C for 48 hours without evidence of infection), UTI (positive pathogen results from urine culture and/or hemoculture positive), sepsis (presence of Sequential Organ Failure Assessment score ≥ 2, and serum lactate level >2 mmol/L), or septic shock (sepsis and persistent hypotension requiring vasopressors to maintain mean arterial pressure ≥ 65 mmHg).^14^^,^^15^

In the surgical supine position, the novel “Giusti’s Position,” as invented and described by Dr. Guido Giusti, was followed.^17^ Ultrasound combined with fluoroscope-guided renal access was favored to reduce radiation exposure, minimize adjacent organ injury, and identify the renal calyx and depth of puncture. In the prone position, fluoroscope-guided renal access (Bullseye technique) was employed.

## Results

### Demographic Data

A total of 290 patients participated, with 87 patients (30%) allocated to the supine group and 203 patients (70%) to the prone group, reflecting an allocation ratio of 1 : 2.3. The overall demographic data of the 2 groups were comparable, except for age, and diabetes mellitus, and multiple tract access, which were higher in the supine group. In contrast, the prone group exhibited higher rates of previous PCNL and preoperative hydronephrosis as presented in Table 1.

The operative time in the prone group was significantly longer than in the supine group (116.3 ± 38.9 vs. 103.9 ± 42.6 min, *P* = .016). However, the SFR was comparable in the 2 groups (83.7% vs. 86.2%, *P* = .383). According to the Modified Clavien-Dindo classification, the incidence of grade 0 complications was more frequent in the supine group (59.7% vs. 35.5%), while grade I complications were higher in the prone group (53.2% vs. 25.3%). Conversely, grade II complications were more prevalent in the supine group (14.9% vs. 4.4%), whereas grade IIIa and IIIb complications occurred exclusively in the prone group (6.4% vs. 0% and 0.49% vs. 0%, respectively) from pseudoaneurysm needs angioembolization in 1 patient, pleural complications, including hydrothorax. They need conservative management, including oxygen therapy in 4 patients, simple needle aspiration in 5 patients, intercostal drainage in 3 patients, and 1 patient required cystoscopy with clot evacuation and ureteral stent insertion under general anesthesia. No patients need Intensive Care Unit (ICU) monitoring, as summarized in Table 2.

Overall complication rates were similar in the 2 groups. However, pleural injury was significantly higher in the prone group (6.4% vs. 0%, *P* = .016). Urological infectious complications were noted with fever in 155 patients (53.44%), UTI in 76 patients (26.21%), sepsis in 41 patients (14.14%), and septic shock in 3 patients (1.03%). These complications occurred more in the prone group with statistically significant differences (*P* < .05) in fever (62.07% vs. 33.33%), UTI (32.51% vs. 11.49%), and sepsis (17.24% vs. 6.90%). Comprehensive data on complications are provided in Tables 2 and 3.

### Perioperative and Postoperative Outcomes

### Risk Factors

Univariable logistic analysis revealed that staghorn calculi, prone position, operative time longer than 90 minutes, and a positive preoperative urine culture increased infection rates. Multivariable logistic analysis confirmed that prone position (RR 4.83; 95% CI 1.26-4.57; *P* = .004) and a positive preoperative urine culture (RR 4.41; 95% CI 2.82-6.89; *P* < .001) were independent risk factors. Detailed results are presented in Table 4.

## Discussion

Based on prior experience, a study of PCNL^18^ performed in the prone position was carried out, and inevitable postoperative infectious complications, including fever (38%) and UTI (25%), were observed. Therefore, supine PCNL was established in the unit in 2018, aiming to mitigate these complications based on the hypothesis of lower intrarenal pressure.^19^

This study demonstrated a significant advantage of the supine position compared to the prone position in reducing postoperative urological infectious complications, including fever (33.3% vs. 62.1%, *P* < .001), UTI (11.5% vs. 32.5%, *P* < .001), and urosepsis (6.9% vs. 17.2%, *P* = 0.021), consistent with current evidence regarding the influence of surgical positioning on infectious complications.^9^ The study revealed several factors associated with an increased risk of postoperative infections, including staghorn calculi (RR 1.51, *P* = .04), operative time longer than 90 minutes (RR 2.26, *P* = .001), the prone position (RR 2.83, *P* = .001), and positive preoperative urine culture (RR 4.18, *P* < .001). Multivariable analysis confirmed that the prone position (RR 4.38, *P* = .004) and positive preoperative urine culture (RR 4.41, *P* < .001) were independent risk factors for postoperative infections. Additional risk factors included stone burdens and a history of UTI.^9^

Recent studies on supine PCNL have demonstrated its efficacy in achieving comparable SFRs to the prone position.^6^^,^^9^^,^^20^^,^^21^ Furthermore, the supine position offers several advantages, including shorter operative times, fewer intraoperative and postoperative complications,^8^ and the ability to perform concomitant procedures such as ECIRS or SBES.^6^^,^^22^ In this study, SFR, blood loss, and blood transfusion requirements were comparable between the 2 positions. However, the supine group showed significantly better outcomes in terms of operative time and pleural complications. It was analyzed that the supine position has a low postoperative infection rate, as lower intrarenal pressure is a key factor in reducing infection rates and improving stone clearance through gravity drainage during the procedure.^9^^,^^23^ Additionally, the reduced operative time may contribute to a lower risk of bacterial translocation into the systemic circulation. In contrast, the higher infection rate in the prone group may be attributed to higher intrarenal pressure, longer operative time, and preoperative hydronephrosis, which may contribute to infection risk. A positive preoperative urine culture, indicating bacterial colonization and urothelial disruption during the procedure, can exacerbate infection risks. This may result from the release of bacterial endotoxin during stone fragmentation and continuous fluid irrigation, which can enter the bloodstream through pyelovenous, pyelolymphatic, and pyelotubular backflows or forniceal rupture.^23^ In the study, postoperative infections were managed following sepsis protocols,^14^^,^^15^ including intravenous antibiotics, fluid resuscitation, and vasopressors if needed; no patients need ICU monitoring. The prophylactic antibiotics reduce the risk of postoperative infection; however, it does not completely eliminate it. Therefore, the preoperative administration of an empirical antibiotics protocol to achieve sterile urine is essential.

This study has several limitations. First, this study is a retrospective study conducted at a single center. Second, the procedures were performed by 2 different experienced surgeons, which may have introduced variability and played a role in the outcome of the supine position. Third, the sample size of patients undergoing PCNL was relatively small. Lastly, the clinical decision-making process for managing infectious complications was individualized, potentially introducing treatment bias.

Future research should focus on conducting multicenter, prospective studies or randomized controlled trials to minimize bias and confounding factors, increase the sample size, and incorporate tools to measure intrapelvic pressure during procedures performed in both supine and prone positions with standard and miniature tract size. Additionally, the use of suction devices should be explored to reduce intrapelvic pressure, improve SFRs, and decrease complications in the prone position. Finally, developing a preoperative antibiotics protocol to treat bacterial colonization until sterile urine is obtained before the operation in patients who are at high risk of infection.

Supine PCNL has a better safety profile with significantly lower postoperative infection rates and high-grade complications compared to the prone position. Additionally, supine PCNL demonstrated shorter operative times, reduced pleural complications, and equivalent efficacy in SFRs, blood loss, and blood transfusion requirements compared to the prone position.

## Figures and Tables

**Table 1.** Demographic Data

Demographic Data	Prone Position (n = 203)	Supine Position (n = 87)	*P*
Age, year, mean (SD)	55.9 (12.7)	60.5 (13.9)	+**.006**
BMI, kg/m^2^, mean (SD)	25.7 (4.6)	26.4 (5.9)	.276
Gender Male, n (%) Female, n (%)	107 (52.71) 96 (47.29)	38 (43.68) 49 (56.32)	.159
Diabetes, n (%)	48 (23.76)	31 (35.63)	+**.038**
Staghorn calculi, n (%)	105 (51.72)	37 (42.53)	.151
Previous PCNL, n (%)	31 (15.27)	6 (6.90)	+**.050**
Maximal stone size, mm, mean (SD)	35.2 (15.9)	31.8 (11.7)	.074
Single renal calculi, n (%)	164 (80.79)	69 (79.31)	.772
Present of hydronephrosis, n (%)	179 (88.18)	58 (66.67)	+**<.001**
Pre-operative nitrite positive, n (%)	29 (14.29)	12 (13.79)	.912
Pre-operative urine culture positive, n (%)	72 (35.47)	27 (31.03)	.466
Mini-PCNL, n (%)	13 (6.40)	11 (12.64)	.102
Multiple tract access, n (%)	11 (5.42)	18 (20.69)	+**<.001**

BMI, body mass index; kg, kilograms; m^2^, square meter; n, number; PCNL, percutaneous nephrolithotomy; SD, standard deviation.

**Table 2.** Peri-Operative and Post-Operative Outcomes

Outcomes	Prone Position (n = 203)	Supine Position (n = 87)	*P*
Operative time, min, mean (SD)	116.3 (38.9)	103.9 (42.6)	+**.016**
EBL, ml, median (IQR)	100 (100-200)	130 (80-250)	.437
Blood transfusion, n (%)	10 (4.93)	7 (8.05)	.300
Stone free, n (%)	170 (83.7)	75 (86.2)	.383
Pleural complication, n (%)	13 (6.40)	0	+**.016**
Modified Clavien-Dindo classification 0 (No complication) I (Fever) II (UTI, or Urosepsis, blood transfusion, intravenous antibiotics) IIIa (Needle aspiration, intercostal drainage, ureteral stenting, embolization) IIIb (VATS or intervention under general anesthesia)	72 (35.47) 108 (53.20) 9 (4.43) 13 (6.40) 1 (0.49)	52 (59.77) 22 (25.29) 13 (14.94) 0 0	+**<.001**
LOS, day, median (IQR)	4 (4-5)	4 (4-5)	.159

EBL, estimated blood loss; IQR, interquartile range; LOS, length of hospital stays; min, minutes; ml, milliliters; n, number; SD, standard deviation.

**Table 3.** SIRS and Infectious Complications

Complications	Prone Position (n = 203)	Supine Position (n = 87)	*P*
Fever, n (%)	126 (62.07)	29 (33.33)	+**<.001**
UTI, n (%)	66 (32.51)	10 (11.49)	+**<.001**
Sepsis, n (%)	35 (17.24)	6 (6.90)	+**.021**
Septic shock, n (%)	2 (0.99)	1 (1.15)	.899

n, number; SIRS, systemic inflammatory response syndrome; UTI, urinary tract infection.

**Table 4.** Univariable and Multivariable Logistic Analysis for Postoperative Infection

Factors	RR (95% CI)	*P*	Adjusted RR (95% CI)	*P*
Age >50 years	1.036 (0.680-1.580)	.866		
DM	1.014 (0.659-1.561)	.946	1.021 (0.772-1.348)	.884
Staghorn renal calculi	1.512 (1.018-2.246)	+**.040**	0.995 (0.741-1.335)	.975
Prone position	2.828 (1.527-5.236)	+**.001**	4.384 (1.263-4.566)	+**.004**
Previous PCNL	0.693 (0.345-1.392)	.304		
Maximum stone diameter >3 cm	1.498 (0.991-2.265)	.055	1.317 (0.961-1.806)	.086
Present of hydronephrosis	0.526 (0.269-1.027)	.060	0.705 (0.402-1.237)	.224
Operative time >90 minutes	2.258 (1.372-3.714)	+**.001**	1.139 (0.836 -1.551)	.407
Pre-operative urine culture positive	4.180 (2.751-6.351)	+**<.001**	4.408 (2.821-6.887)	+**<.001**
Mini-PCNL	0.615 (0.246-1.539)	.300		
Multiple tract access	1.208 (0.676-2.160)	.522		
Blood transfusion	1.629 (0.890-2.979)	.113	1.118 (0.686-1.822)	.654

DM, diabetes mellitus; PCNL, percutaneous nephrolithotomy.

## Data Availability

The data that support the findings of this study are available on request from the corresponding author.
